# Experiments On Sublimating Carbon Dioxide Ice And Implications For Contemporary Surface Processes On Mars

**DOI:** 10.1038/s41598-017-14132-2

**Published:** 2017-10-27

**Authors:** L. E. Mc Keown, M. C. Bourke, J. N. McElwaine

**Affiliations:** 1 Department of Geography, School of Natural Sciences, Trinity College Dublin, College Green, Dublin 2, Ireland; 2 Planetary Science Institute, 1700 E Fort Lowell Rd, Tucson, 85719 AZ USA; 3 Department of Earth Sciences, Durham University, Durham, DH1 UK

## Abstract

Carbon dioxide is Mars’ primary atmospheric constituent and is an active driver of Martian surface evolution. CO_2_ ice sublimation mechanisms have been proposed for a host of features that form in the contemporary Martian climate. However, there has been very little experimental work or quantitative modelling to test the validity of these hypotheses. Here we present the results of the first laboratory experiments undertaken to investigate if the interaction between sublimating CO_2_ ice blocks and a warm, porous, mobile regolith can generate features similar in morphology to those forming on Martian dunes today. We find that CO_2_ sublimation can mobilise grains to form (i) pits and (ii) furrows. We have documented new detached pits at the termini of linear gullies on Martian dunes. Based on their geomorphic similarity to the features observed in our laboratory experiments, and on scaling arguments, we propose a new hypothesis that detached pits are formed by the impact of granular jets generated by sublimating CO_2_. We also study the erosion patterns formed underneath a sublimating block of CO_2_ ice and demonstrate that these resemble furrow patterns on Mars, suggesting similar formation mechanisms.

## Introduction

The Martian atmosphere, which is comprised of over 95% CO_2_^1^, interacts seasonally with the planetary surface. As temperatures fall between late autumn and early winter, a CO_2_ deposit covers the surface^2^ in thicknesses ranging from around a metre in the polar regions^1,3^ to a few millimetres towards the equator^4^. The distribution of this dry ice is governed primarily by solar insolation^5^. In the early spring, as insolation increases, the dry ice begins to sublimate. This process is now recognised as an important agent in the formation of contemporary surface features on Mars. These features include the dendritic araneiform terrain of the south polar cryptic region^6–8^, linear gullies, their associated pits^9,10^, and sand furrows^11^. In this study we focus on linear gully pits and sand furrows; both active features which are observed to form and evolve on dunes under the current Martian climate. These features have no Earth analogues and the specific mechanisms responsible for their formation have yet to be fully understood and quantified.

## Furrows

**Figure 1:**
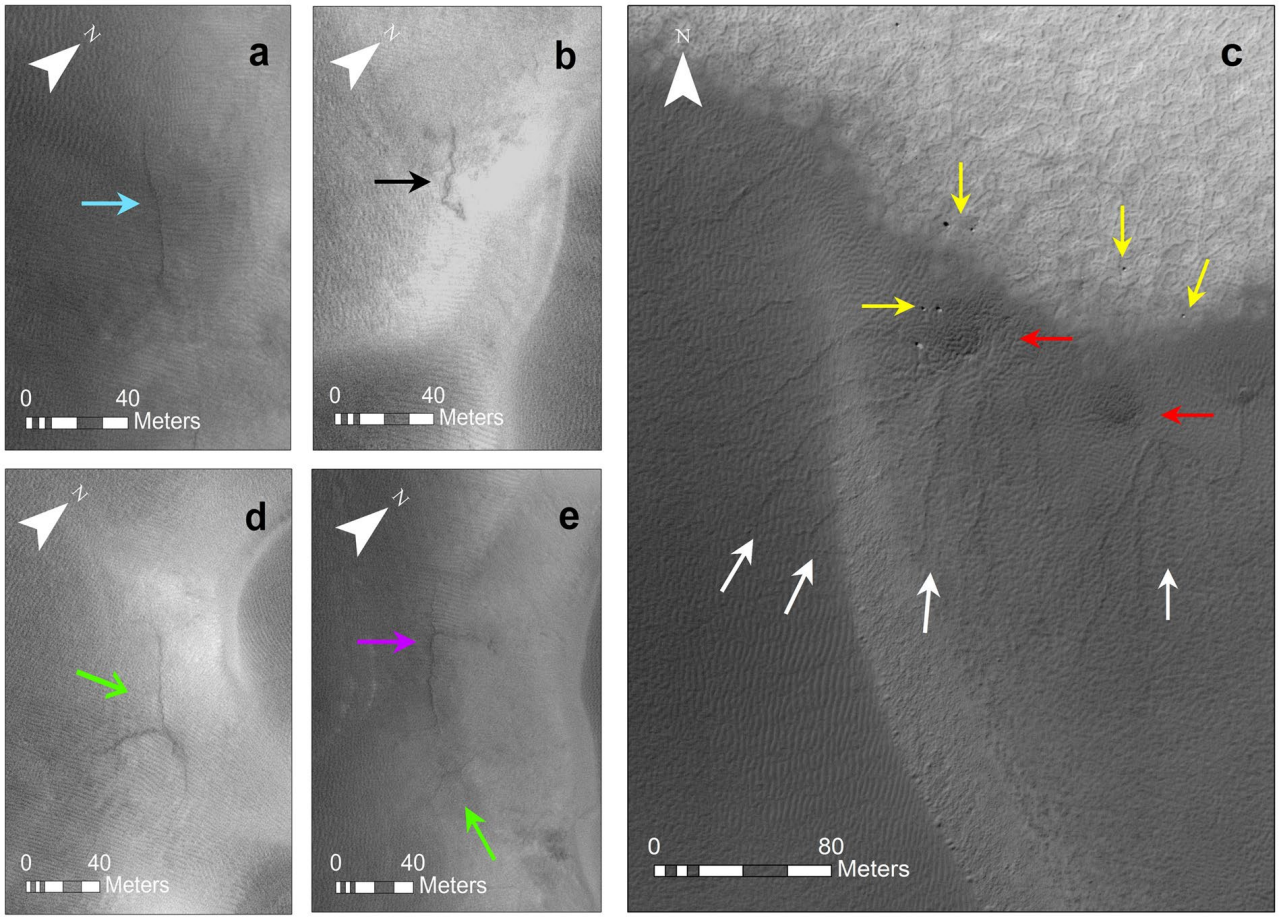
Examples of sand furrows on Martian Dunes. (**a**) Linear furrow (blue arrow) on sand dune in Chasma Boreale (all Chasma Boreale furrows are taken from HiRISE image ESP_026851_2590, latitude 78.65°, longitude 308.494°, solar incidence angle 56° with the Sun ~34° above the horizon). (**b**) sinuous (black arrow) furrow on sand dune in Chasma Boreale, (**c**) dendritic furrows (white arrows) on sand dunes at latitude −67.607°, 185.343° in the southern hemisphere (HiRISE image ESP_023270_1120. Solar incidence angle is 60° with the sun ~30° above the horizon). Yellow arrows point to boulders, red arrows point to dark fans and highlight their proximity to the furrows. (**d**) radial (green arrow) furrow on sand dune in Chasma Boreale. (**e**) rectilinear (purple arrow) and radial (green arrow) furrows on sand dune in Chasma Boreale. Images have been stretched to improve contrast as furrows are narrow, shallow and thus subtle features. HiRISE image credit: NASA/JPL/University of Arizona.

Furrows are shallow (~0.25 m) and narrow (~1.5 m) negative relief^11,12^, features which are observed on over 95% of the northern polar dunes^11^ and are also found between 40°S and 72°S^13^. Their network patterns range from rectilinear, to dendritic and radial, tributary and distributary and their planforms can be linear or sinuous^12^ (Fig. 1), though it is unclear what factors control this variety of patterns. Appearing to “defy gravity”, furrows extend upslope and transect existing aeolian ripples, and so while their patterns may resemble those eroded by fluvial activity on Earth, gravity-driven processes are unlikely to form them^12^.

### Furrow Formation Hypotheses

Furrows frequently form after the appearance of polygonal cracks in CO_2_ ice. This led Bourke^12^ to hypothesise that they were caused by cryoventing; that is, basal sublimation of CO_2_ and consequent erosion. This is similar to Kieffer’s model for the dark spots and fans in the southern hemisphere^14^, but applied in the context of dunes. The cryoventing model proposes that in the spring, gas pressures increase at the ice-substrate interface until the overlying ice cracks. Gas rapidly exits, eroding material below the ice sheet to form furrows. A plume then deposits the sediment to form fans on the surface of the seasonal ice. The thickness of the overlying ice and dune topography are thought to have a strong influence on this process^15^. Close spacing of vents has also been noted to reduce cryoventing efficacy, with fewer furrows forming in locations where ice cracks are closer together^12^.

Recent work has drawn a distinction between furrows in the northern hemisphere and “dendritic troughs” in the southern hemisphere^16^. While both show similar morphologies, furrows in the northern hemisphere are ephemeral features which disappear every year^17^. The dendritic troughs on south polar dunes endure, with new tributaries adding to the networks annually^16^. This study^16^ has drawn a potential link in formation process between the highly dendritic araneiform terrain of the south polar cryptic region and furrows/dendritic troughs. It has been suggested that araneiform terrain (dubbed “spiders” in the literature) may develop over many years by a gradual connection of dendritic networks into a radial network, rather than forming in one event^16^. Noting the difference in environmental conditions between the hemispheres, it has been argued that furrows in the northern hemisphere do not develop into dendrites because of the high mobility of the material into which they are eroded^16^. However, the size distribution of the granular substrate in both hemispheres is poorly constrained. For the purpose of our purely morphological laboratory study we refer to all network types as furrows.

Recent experimental work has demonstrated that insolation-driven dust ejecta from within CO_2_ ice is a viable process^18^ and that pressure driven processes can form dendritic patterns under certain conditions^19^. However, it has not yet been demonstrated that sublimating CO_2_ in contact with porous substrate can transport underlying material and create the complex furrow morphologies seen on Mars. Until now, this geomorphic process has been framed in a purely theoretical context.

## Linear Gully Pits

**Figure 2:**
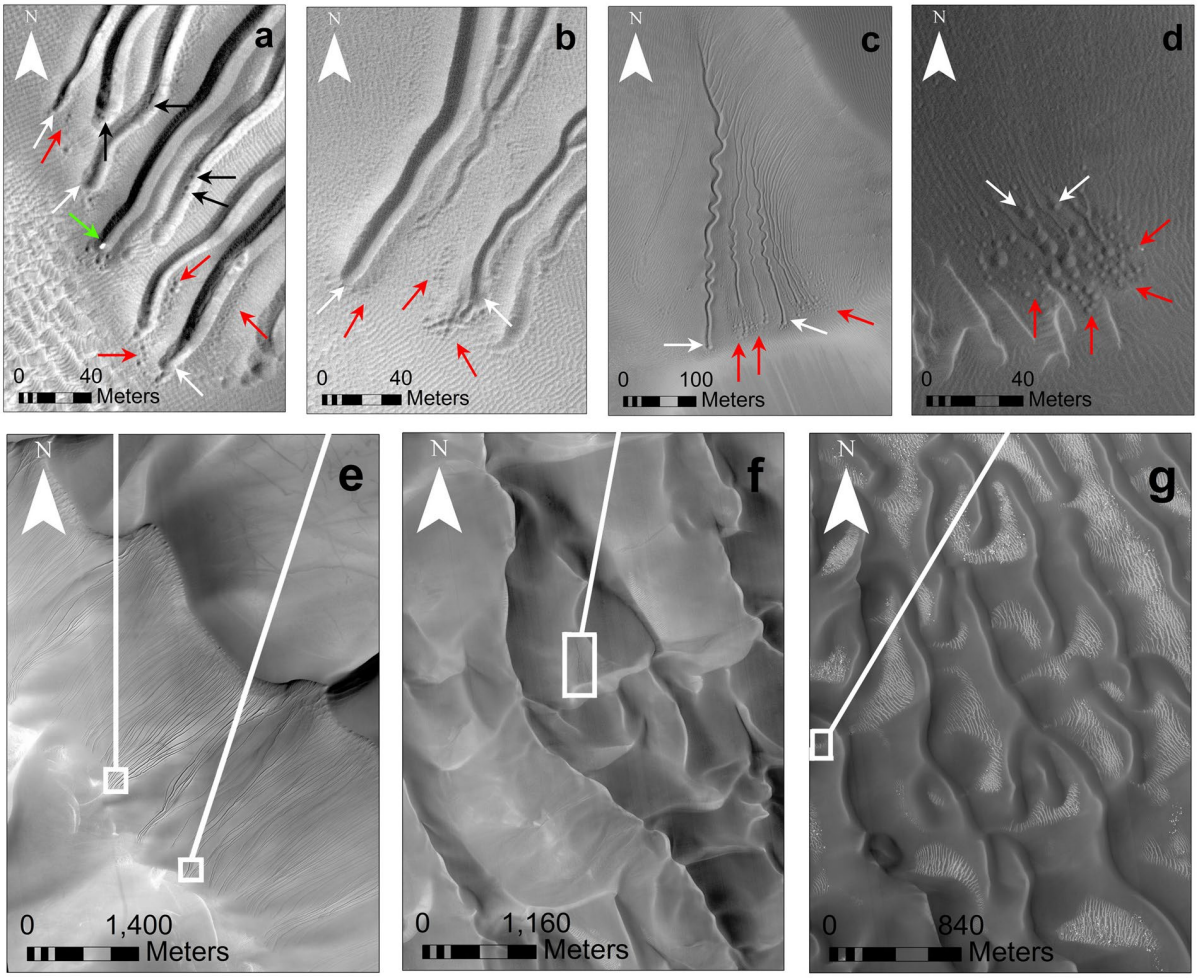
Terminal and detached linear gully pit morphologies. (**a**) Terminal pits (white arrows), and detached pits (red arrows) at linear gully termini on Russell Crater megadune (HiRISE image ESP_020784_1255, image centre at −54.3°, 12.9°, taken at L_*s*_ 209.5°). Black arrows point to inset pits within terminal pits. Green arrow indicates a high albedo block which is likely to be a CO_2_ block within a pre-existing terminal pit. (**b**) Terminal pits (white arrows) and detached pits (red arrows) on Russell Crater megadune (HiRISE image PSP_007018_1255 taken at L_*s*_ 22.5°, image centre at −54.3°, 13° which was used as part of a stereo pair to create the DTM, DTEEC_007018_1255_007229_1255). (**c**) Example of highly sinuous linear gullies on the west side of Matara Crater dunefield (HiRISE image ESP_030528_1300, image centre at −49.5°, 77.2°, taken at L_*s*_ 254.8°). Red arrows point to detached pits, white arrows show terminal pits. (**d**) “Tadpole” terminal pits in Proctor Crater dunefield (HiRISE image PSP_003800_1325, image centre at −46.9°, 30.7°, taken at L_*s*_ 240.9°). These pits (white arrows) are much wider in diameter than their corresponding channels and are surrounded by detached pits (red arrows). (**e**) Russell Crater megadune (HiRISE image PSP_007018_1255).  White boxes indicate locations of **(a)** and** (b)**. (**f**) Matara Crater dunefield. White box shows location of (**c**). (**g**) Proctor Crater dunefield (HiRISE image PSP_003800_1325). White box shows location of (**d**). HiRISE image credit: NASA/JPL/University of Arizona.

Linear gullies (Fig. 2) form on Martian dunes under current climatic conditions^10,20–22^. Recent mapping has shown that they occur between 36.3°S and 54.3°S and from 64.6°S to 70.4°S^22^. They are characterised by long (100–2,500 m), narrow grooves, are bounded by levées and have relatively small source areas. Forming exclusively on south polar and mid latitudinal intra-crater dunes, their activity is seasonal, and has been found to be concurrent with the final stages of sublimation of the CO_2_ ice deposit at the end of winter and beginning of spring^22^, rendering a CO_2_ sublimation formation mechanism likely. These eponymous features are mostly linear; though they sometimes adopt sinuous patterns and taper down-slope.

### Terminal pits

Unlike terrestrial gullies, which are nearly always formed by liquid water, linear gullies do not have associated debris aprons. Instead, their channel termini are invariably punctuated by one (or more) terminal pit(s) (Fig. 2a–d). Located at the end of the linear gully channel, the terminal pit is usually slightly wider than the channel^10^. Terminal pits are defined by a roughly circular depression encircled by levées. Most linear gully channels end in a single pit, herein referred to as the primary terminal pit. However, some terminal pits occur as part of a dyadic to triadic sequence known as “pit strings”. Secondary and tertiary terminal pits are defined as terminal pits that are in a linear sequence with the primary terminal pit, and are always similar in size to the primary terminal pit. Many linear gullies, particularly the largest in scale, have lower albedo circular depressions *within* the distal region of their channels and/or their primary terminal pits. We refer to these as inset pits.

### Detached pits

Some terminal pits or terminal pit strings are surrounded by multiple detached pits (Fig. 2a–d). These pits are not connected to (or in line with) channels and are usually considerably smaller than their associated terminal pit(s). Detached pits are always exclusive to the distal region of the channel and are not found upslope.

### Pit Formation Hypotheses

Linear gully pits have no terrestrial analogues, and so it is likely that a geomorphic agent that does not occur naturally on Earth may be instrumental in their formation. Originally, terminal pits were proposed to be older flow fronts formed by successive debris flows^20^, however this is unlikely as linear gullies are forming today, in a climate which does not currently support liquid water^10^.

There are two main schools of thought on the type of CO_2_ sublimation activity which might form linear gullies: the CO_2_ basal sublimation triggered debris flow hypothesis^23^ and the sliding CO_2_ block hypothesis^21^. The CO_2_ basal sublimation triggered debris flow hypothesis^23^ suggests a solar-insolation-driven dry debris flow beneath seasonal CO_2_ ice on dunes. Successive events have been suggested to carve gully channels in Russell Crater^23^. However, while basal sublimation — driven debris flows are a plausible mechanism for channel formation, such a process would also deposit material at channel termini. Additionally, the model does not account for the presence of terminal pits or detached pits. The sliding CO_2_ block hypothesis^21^ is currently the only process that can account for the formation of channels and terminal pits which distinguish linear gullies from other gully morphologies, making this class of gullies exclusive to Mars. The hypothesis proposes that in spring, ice blocks detach from dune brinks. The blocks then descend dune slopes supported by a lubricating layer of CO_2_ gas. This gas layer is engendered by a mechanism similar to the Leidenfrost Effect^24^, which occurs when a liquid or solid is in thermal contact with a surface that is at a temperature far beyond its boiling or sublimation point. Upon surface contact, the substance (in this case, CO_2_ ice), will levitate on a cushion of gas, because the total force exerted by the gas pressure under the block exceeds its weight. This lubricating gas layer eliminates, or reduces, frictional forces so that blocks may move down even very gentle slopes of a few degrees. As the block moves it will carve out channels and deposit lateral levées, until it eventually stops to sublimate at the terminus. This ultimate sublimation explains the formation of a terminal pit. The block is proposed to rest at the base of the channel while sublimating, eroding a depression beneath it and transporting material to the side to form levées. This protracted stationary sublimation may explain why pits are commonly slightly wider than their corresponding channels, which are proposed to form by the rapid movement of blocks downslope^21^.

Thus far, alcove, channel, levée and shallow pit morphologies have been observed to form when CO_2_ ice blocks were slid onto sand dunes in Utah and Arizona^25,26^. Further evidence consistent with this hypothesis was the detection of high albedo blocks within linear gully channels on Mars. These are most likely CO_2_ ice blocks “caught in the act”, of sliding through pre-existing linear gully channels^10,21^.

It is possible that secondary and tertiary terminal pits may form by blocks that bounce at the channel terminus in the manner observed during field tests on sand dunes in Utah^26^. It has also been proposed that the multiple detached pits which surround many linear gully termini may be formed by smaller parts of blocks which have broken up at the terminus, scattering ice fragments into their surroundings^21^. However, the formation mechanism responsible for pronounced terminal pits and detached pits remains uncertain. It is not known if a stationary CO_2_ block can erode and deposit material to form the full range of pits identified here. Why detached pits are exclusive to a small fraction of linear gullies and why they are restricted to the lower part of the channel is also not yet clear. If detached pits were formed by broken blocks, it is conceivable that blocks would also break at locations such as sharp bends in the channel, areas of dune surface undulation and locations where channels merge. These are all regions where a block would be subjected to stress and yet detached pits are only found at channel termini. We hypothesise that if stationary sublimating CO_2_ blocks have the geomorphic agency to excavate and deposit material to form pits and circular levées, they must undergo a rapid sublimation process to do so. We reason therefore that blocks which reach the terminus may emit CO_2_ jets as they sublimate. If these jets entrain sufficient granular material^27^ they may then be capable of eroding detached pits when they return to the surface.

Prior to this study, measurements of linear gullies has concentrated on geographical trends^22^ and the linear gully system as a whole^21,26^. Comprehending pit formation is crucial to understanding the formation mechanism responsible for this extra–terrestrial gully type. Therefore, it is important that we test whether sublimating CO_2_ ice blocks can form pit morphologies.

## Methods

### Objectives

To test the CO_2_ block hypothesis, in the context of pit formation, we first conducted a short survey of Martian linear gully terminal pits and detached pits on dunes in Russell, Proctor and Matara Craters. The objectives of this study were to (i) characterise the different terminal pit morphometries, (ii) investigate if there was any evidence of a link between these terminal pit morphometries and detached pits and (iii) investigate the range of block sizes which may be reactivating and widening linear gully channels seasonally.

To further test the CO_2_ hypothesis, in the context of pit formation, we performed experiments to examine whether sliding and hence partially buried CO_2_ ice blocks would form morphologies comparable to Martian linear gully primary terminal pits and detached pits.

To test the cryoventing hypothesis, in the context of furrow formation, we performed a second set of experiments with two aims: (i) to study the erosion patterns resulting from the interaction between a gently placed CO_2_ block and granular substrate and compare these features to a pre-existing Martian furrow network classification^12^; (ii) to study the factors which constrain furrow pattern type and furrow density.

### Survey of Linear Gully Pits in Proctor, Russell and Matara Craters

High Resolution Imaging Science Experiment (HiRISE) Digital Terrain Models (DTMs) DTEEC_003080_1325_004077 and DTEEC_007018_1255_007229 and corresponding orthophotos were employed to survey linear gully pits in Proctor Crater and Russell Crater, respectively. The polygon tool in ArcMap 10.4 was used to measure the area of individual terminal pits (along the levée apex), taking the diameter of this circle as the pit width. Terminal pit area was calculated by squaring the pit radius and multiplying by *π*. Pit depths were measured using the Interpolate Line tool, by drawing a line across the pit centre and extending this to the surrounding dune surface. Depth was estimated as the difference in elevation between a pit floor and the surrounding dune surface on each side and averaging these values.

The Russell Crater DTM (DTEEC_007018_1255_007229) has an estimated vertical precision of 1.2 m^28^. The relatively low estimated vertical precision is attributed to a low convergence angle (or sum of emission angles) between the stereo pair used to develop the DTM, which reduces vertical precision. Horizontal accuracy of this DTM was given by post spacing, which was 1 m/pixel. The Proctor Crater DTM (DTEEC_003080_1325_004077) has an estimated vertical precision of <0.5 m^28^ and a horizontal accuracy of 1 m/pixel. Pit widths in both locations were generally much wider than 1 m and so horizontal accuracy should not significantly affect our measurements. Both sites were dark dunes and so noise in the DTMs may have affected our measurements to a small degree. We have estimated an upper limit of this effect by taking 10 linear cross sections close to pit locations in both DTMs. We detrended these cross sections, averaged them and calculated the standard deviation as an estimate of noise^29^. In Russell Crater this value was 1.7 m and in Proctor Crater, the value was 1.08 m and so depths <1.7 m were not reported in Russell Crater and depths less than 1.08 m were not reported in Proctor Crater. Horizontal measurements from orthophotos at both locations may have been affected by atmospheric dust and detector noise which was at the pixel level and would affect our horizontal measurements by one pixel at most.

A time-series of HiRISE images was used to determine whether there may be a link between detached pit and terminal pit formation. We propose that larger pit sizes (that indicate larger ice blocks) have a higher probability of generating detached pits. Terminal pit areas were measured as outlined above and the number of detached pits surrounding them were counted. Detached pits were identified as low albedo depressions. Negative topography was confirmed using the Interpolate Line tool to generate topographic profile data across the feature in the corresponding DTM where possible. Smaller features that may have been artefacts of dune surroundings (*e.g*. shadows in ripples), were not included. HiRISE images taken at the same location (−54.3°, 12.9–13°) on the Russell megadune and Matara Crater dunes (−49.5°, 34.7° and −49.5°, 34.6°) allowed us to identify new or widened terminal pits and new detached pits. Suitable data were not available for Proctor Crater.

The Russell Crater observations ranged over 4 Mars years (MY) between MY 29 and 32. The images used were ESP_012213_1255, ESP_020784_1255, ESP_029764_1255 and ESP_039153_1255 for MY 29, 30, 31 and 32 respectively. These images have emission angles of 8.2°, 5.1°, 3.8° and 3.8° respectively. Emission angle is the angle between the HiRISE camera and a normal drawn perpendicular to the surface, where 0° is known as *nadir*). Roll directions (obtained by comparing image centre longitude and subspacecraft longitude) were from west, east, east and west, respectively. For Matara Crater the survey extended over 2 MY between MY 30 and 31 (HiRISE images ESP_020414_1300, ESP_029750_1305 for sites at −49.5°, 34.7°. These images have emission angles of 4.7° looking from west and 0.4° looking from east respectively. ESP_021759_1300 and ESP_030528_1300 were examined for sites at −49.5°, 34.6°. These images had emission angles of 9.7° looking from east and 12.2° looking from east, respectively. Differences in lighting were accommodated for by adjusting contrast and brightness in the overlapping images. New detached pits were identified as circular depressions of low albedo that were not in the previous MY image and which surrounded a terminal pit. The extent to which pits were widened (if any) was measured by fitting a circular polygon to the same terminal pit for two consecutive Mars years, calculating area as outlined above, and differencing these data. Early spring images were examined for each location in order to measure high albedo features thought to be CO_2_ blocks within channels. This was done by zooming in to optimal pixel resolution and using the Measure tool to record their width and length.

The images were taken at similar *L*_*s*_, or solar longitude (the Mars/Sun angle, measured from the northern hemisphere spring equinox where *L*_*s*_ = 0, a measurement used to quantify Martian seasons). Images were also selected based on the emission angle. To minimise the effect of geometric distortions, single colour RDR images were used in each case. These are radiometrically-corrected images which are map projected. The radiometric correction adjusts for instrument offset, dark current and gain and then converts pixel values to $\frac{Intensity}{Flux}$ reflectance. Geometric processing corrects for optical distortion and projects the image from spacecraft viewing to a map coordinate system. The MOLA (Mars Orbiter Laser Altimeter) DTM is used to improve the camera pointing intercept position on the Martian surface. Orthorectification corrects for distortions that may occur in off-nadir images where the spacecraft roll angle causes pixel foreshortening in the cross-track direction^30^. The images we used were not orthorectified, and so disparities may occur when comparing images with different observation geometry. To minimise this effect, images close to nadir were chosen and care was taken to select images with less than a 5° difference in emission angle. Because such differences are small, we can neglect parallax distortions^31^. A correction was made for any minor deviations however by dividing *x*-direction measurements by the cosine of the emission angle^30^. In each case the distortions are within tens of centimetres and thus fall within errorbars for our measurements.

### Experimental Setup

In order to investigate the CO_2_ block hypothesis for pit formation^21^ and the cryoventing hypothesis for furrow formation^11^ we performed laboratory experiments. Initial pilot work, under ambient terrestrial conditions, revealed that water in the atmosphere had a significant effect as it formed frost on the surface of the block and on the bed. This affects the heat budget, the permeability of the bed and the mobility of the grains and must be avoided. Additionally, this frost would later melt and erase the surface microtopography. Therefore, we performed our experiments in a low humidity chamber. The chamber was erected on a level surface in a constant temperature (Δ*T* ≈ 3 K) laboratory. A plastic container (460 × 675 × 400 mm) was filled with dehumidifying silica gel beads. A smaller plastic container (300 × 520 × 370 mm) was placed inside, forming a silica gel bead moat which surrounded the interior container. A perspex lid was fitted on top of the chamber and vacuum bagging gum sealant tape was added at the interface between the container and lid to ensure the chamber was air-tight. A sealed trap door was constructed within the perspex lid in order to minimise exposure to atmospheric water vapour when placing blocks inside.

Prior to each experimental run, three CO_2_ ice blocks were placed upon the silica bead moat and were given time to sublimate. These generated dense CO_2_ gas which flushed out the original gaseous content, thus removing any water vapour. This reduced the relative humidity sufficiently so that there was no noticeable frost formation during the experiments on the ~−80 °C CO_2_ ice blocks.

Though grain sizes at linear gully and furrow locations on Mars have not been constrained, we used the preliminary data collected by the Curiosity Rover in the Bagnold dune setting on Mars to optimise the range of grain sizes employed in our experiments. We estimated a scale factor of 0.61 (see *Supplementary Material, Experimental Scaling*) by which to reduce grain size to compensate for the disparity in gravity between Mars and Earth. Grain sizes detected in the Bagnold dunes ranged from fine to coarse sand^32^, with many passing through a <150 *μm* sieve. The average grain size detected in the Bagnold dunes was between 200 and 300 *μm* ^32^. When scaled, these ranges fall between <90 *μm* and 122–183 *μm*, respectively. Therefore, Guyson Honite Glass Spheres of four grain diameter ranges (4–45 *μm*, 45–90 *μm*, 75–150 *μm* and 160–212 *μm*) were used for sixteen separate experimental runs.

### Experimental Protocol

In the low humidity environment, pure CO_2_ ice blocks were slid onto beds of glass spheres of each grain size range. We define “sliding” as a gentle motion nudging the block onto the bed surface — sliding is a motion which has enough force to slightly disrupt the granular surface. Sublimation then transports the grains underneath and near the edge of the block. We used Structure from Motion^33^ (SfM) to build Digital Elevation Models (DEMs) of the resultant morphologies from each experiment. We then compared these morphologies and morphometries with Martian terminal and detached pits measured using HiRISE images and DTMs.

A second set of experiments was designed to study the formation of furrows. The blocks were placed as gently as possible on a flat granular bed in order to generate CO_2_ gas flow beneath the block. The aim was to investigate whether such a layer of gas at the interface between CO_2_ ice and a granular substrate could form furrow networks on an initially smooth and level bed. SfM was again employed to build high resolution DEMs of the features produced and the resulting furrow morphologies were compared with the well-characterised furrow networks on Martian dunes^11,12^.

Each granular sample was dried and sieved to disaggregate material prior to each experiment. The sample was then poured into the inner container and levelled using a spirit level. A time-lapse camera was positioned inside the chamber to record sublimation rate. A digital hygrometer placed on top of the bed indicated depression of relative humidity in real-time. Once relative humidity decreased sufficiently, the trap door was opened and a CO_2_ ice block of mass <1 kg (Table 1) was either placed or slid onto the bed. The chamber was immediately sealed and the block in each case was allowed to sublimate and interact with the granular substrate. This sublimation process lasted ~7–11 hours for each block depending on its mass and whether it burrowed. Videos of the initial sublimation dynamics in each case were recorded with an iPhone 6S 12 megapixel camera from outside the chamber, in order to avoid accumulation of grains on the lens which would hamper video quality.

**Table 1:** Laboratory experiment controlled and measured parameters:

		Dura-tion (hr:min)	DEM reso-lution (cm/pixel)	Ortho-photo reso-lution (cm/pixel)	SfM RMS repro-jection error (pix)	DEM Noise Uncer-tainty (cm)	DEM Hori-zontal Uncer-tainty (cm)	DEM Vertical uncer-tainty (cm)	Rela-tive humi-dity (%)	Gas tempe-rature (°C)	Block mass (g)	Block thick-ness (cm)	Pit depth (cm)	Block length (cm)	Pit length (cm)	Block width (cm)	Pit width (cm)	Max levée height (cm)	Min levée height (cm)	Pre-dicted pit width (m)	Pre-dicted pit length (m)	Predicted pit depth (m)	Pre-dicted pit depth error (m)	Furrow type
		t	*R*_DEM_	*R*_Ortho_	*E*_*RMS*_	*E*_*Noise*_	*E*_*Horiz*_	*E*_*Vert*_	*RH*	*T*_atm_	*M*	*H*_b_	*H*_pit_	*L*_b_	*L*_pit_	*W*_b_	*W*_pit_	*LH*_max_	*LH*_min_	*W*_norm_	*L*_norm_	*H*_norm_	*E*_norm_	
**Exp**.		**Experimental Series 1**																						
**4–45**	**placed**	10h45	0.087	0.011	0.293	0.038	0.079	0.067	12.3	24.2	544	1.5	1.10	19.0	21.8	10.2	14.9	13.4	10.8	1.46	1.15	0.733	0.107	Dendritic, curvilinear
**45–90**	**placed**	10h48	0.108	0.013	0.240	0.038	0.082	0.100	10.0	23.4	680	1.9	0.66	19.3	21.5	12.0	15.0	9.7	5.7	1.25	1.11	0.347	0.116	Sinuous, linear
**75–150**	**placed**	9h35	0.101	0.013	0.136	0.020	0.074	0.168	8.9	24.0	832	2.1	0.50	20.0	22.8	12.2	14.1	5.0	2.8	1.16	1.14	0.238	0.052	Dendritic, linear
**160–212**	**placed**	9h20	0.124	0.016	0.444	0.080	0.071	0.083	5.6	21.7	626	2.0	0.30	20.0	21.0	12.5	13.5	5.3	3.2	1.08	1.05	0.150	0.040	Dendritic, linear
**4–45**	**slid**	8h00	0.075	0.009	0.261	0.024	0.070	0.209	10.2	24.9	803	2.0	2.0	19.8	32.0	11.1	19.1	17.4	5.4	1.72	1.62	1.000	0.130	None
**45–90**	**slid**	9h10	0.080	0.010	0.256	0.100	0.070	0.210	9.9	24.7	813	2.2	1.4	20.2	22.6	12.5	14.8	10.3	6.0	1.18	1.12	0.636	0.106	None
**75–150**	**slid**	9h00	0.083	0.010	0.083	0.038	0.083	0.049	2.7	19.1	761	2.3	0.45	19.8	23.1	11.8	13.4	5.3	3.2	1.14	1.17	0.194	0.027	Sinuous
**160–212**	**slid**	9h40	0.104	0.013	0.293	0.063	0.073	0.068	6.0	24.1	811	2.3	0.41	20.0	23.6	12.2	14.3	4.0	3.0	1.17	1.18	0.179	0.033	None
**Exp**.		**Experimental Series 2**																						
**4–45**	**placed**	10h17	0.064	0.017	0.350	0.031	0.078	0.230	13.3	22.3	818	2.2	1.14	19.5	21.6	12.0	14.0	15.0	1.0	1.17	1.11	0.304	0.114	Sinuous, linear
**45–90**	**placed**	11h25	0.058	0.015	0.770	0.083	0.060	0.290	9.8	20.0	788	2.2	1.33	19.5	23.9	10.6	14.6	9.0	8.0	1.38	1.23	0.600	0.135	Sinuous
**75–150**	**placed**	10h36	0.065	0.018	0.490	0.068	0.080	0.270	3.0	23.8	712	2.2	0.73	20.0	22.2	11.5	13.9	5.5	1.5	1.21	1.11	0.332	0.157	Linear
**160–212**	**placed**	7h35	0.064	0.016	0.520	0.035	0.080	0.050	1.0	23.0	626	2.0	0.49	19.2	20.1	11.5	12.4	5.0	3.6	1.08	1.05	0.245	0.117	Dendritic, linear
**4–45**	**slid**	7h00	0.053	0.013	0.270	0.043	0.079	0.120	13.0	23.2	932	2.5	2.50	20.0	34.2	12.0	19.2	16.5	5.0	1.59	1.71	1.000	0.134	None
**45–90**	**slid**	8h15	0.110	0.013	0.270	0.092	0.092	0.230	1.0	20.7	726	1.7	1.15	19.5	29.5	12.0	16.7	12.5	3.5	1.39	1.51	0.676	0.082	None
**75–150**	**slid**	8h00	0.059	0.015	0.260	0.081	0.086	0.090	12.3	21.2	782	2.5	0.82	19.5	21.7	12.0	14.1	3.9	1.3	1.18	1.11	0.328	0.095	Dendritic, linear
**160–212**	**slid**	9h18	0.138	0.016	0.280	0.043	0.076	0.080	7.1	20.5	832	2.5	0.29	20.0	26.2	12.0	13.8	3.5	1.3	1.15	1.31	0.119	0.033	Linear

Column 1 represents the grain size range used in each experiment and whether the block was placed or slid onto the granular surface. Duration is the time taken for the block to sublimate fully. In the cases where a block submerged, the end of the experiment was taken as the time when the primary pit was observed to cease sinking. SfM RMS reprojection error is the root mean square reprojection error for the markers calculated over all photos where the markers are visible. Low reprojection error is indicative of good localisation accuracy of point projections within the model. DEM noise uncertainty denotes errors measured by taking 5 closely spaced linear profiles on a nominally flat region of the granular surface, detrending the vertical profiles and taking the standard deviation. DEM horizontal uncertainty denotes the uncertainty on horizontal measurements as a result of imprecision in target constancy, estimated via a bootstrap method within the SfM models. DEM vertical uncertainty denotes our upper limit vertical uncertainty estimates for any vertical distortions caused by some photos taken at a perpendicular angle to the viewing subject. This error estimate was derived by taking 10 vertical transects along a 1.3 cm section of a ruler in experiment 1 and a 3.3 cm section of the experimental container close to the region of interest in experiment 2, calculating the average of these values and expressing the uncertainty as the difference between the average vertical measurements observed in the model and 1.3 cm or 3.3 cm respectively. Predicted pit dimensions are normalised pit dimensions calculated as the ratio of pit dimensions to corresponding block dimensions. DEM vertical uncertainty was then propagated along with a block measurement uncertainty of 0.2 cm, to get the uncertainty on predicted pit depth (see *Supplementary Material, Digital Elevation Model Uncertainty Estimates*, for further detail).

### Digital Elevation Model Development

All features resulting from CO_2_ sublimation were modelled in three dimensions by SfM^33^ using Agisoft Photoscan. SfM is a technique for reconstructing three dimensional structures from two dimensional image sequences. Agisoft Photoscan is commercially available software which can photogrammetrically process digital images to create 3D spatial data. Each feature produced was imaged at many overlapping positions. In order to establish scale in the DEMs, coded markers were placed within the scene. Agisoft Photoscan finds the exact centre of coded markers enabling the production of highly accurate DEMs and the accurate measurement of features in the scene. Agisoft recommend that three or more scale bars are optimal. Therefore, a local coordinate system composed of three coded markers at known distances apart from one another, was used for scale definition^34^ to develop our 3D models. This local coordinate system was composed of three black and white circular 12-bit coded markers which were printed on three 6.8 × 8 cm sheets of paper. The centres of these markers were positioned on a flat wooden triangle (of 75 cm^2^ area) and the markers themselves were laminated with a thin layer of plastic^34^. The (*x*, *y*, *z*) coordinates of the marker centres were carefully measured with an Engineer’s Scale prior to placement in the scene^34^ and these were later entered in Agisoft Photoscan to develop scale bars for reference within the models. These coordinates (in metres) were: (0, 0, 0), (0.064, 0.115, 0) and (0.131, 0, 0) and these have accuracy <1 mm. The scale was in a constant location relative to the experimental chamber in each case, the centre of the nearest target on the scale was 15 cm from the chamber. This was close enough so that it could be seen in multiple overlapping images. This served as a reference for scale definition and also helped the processing tool to align images accurately. Constancy was assured by the remote nature of the laboratory — external vibrations were minimised. Care was taken when moving around the region of interest not to cause vibrational disturbances. In order to ensure a vertical orientation of the *z*-plane, the local coordinate system was placed flat on the laboratory bench during each experiment. The planar arrangement of the coded markers was confirmed using a spirit level to ensure the bench was level and the laminate nature of the markers ensured they did not bend.

The images were captured at a maximum distance of ~1 m from the bed surface and minimum distance of ~0.05 m at a variety of angles with respect to the image subject in each case. Camera positions were not recorded, as Agisoft Photoscan can compute accurate estimates. The focal length on the camera and aperture were fixed at 4.15 mm and f2.2 respectively and otherwise, the camera was not calibrated. Between 41 and 100 images were captured for each experiment, depending on whether fine detail such as furrow patterns were to be captured, or whether primary pit dimensions alone would be measured. The images were then aligned and referenced in Agisoft Photoscan, to build a point cloud, mesh and generate a DEM and corresponding orthophoto of each feature (resolutions in Table 1). The dimensions of each pit were then measured in ArcMap 10.4, using the DEM and orthophoto.

Differencing before and after DEMs in order to estimate pit depth was not possible due to the high albedo of the initial flat bed. We approximated the initial level surface by taking an average of 5 linear cross sections of the flat bed surrounding (but farthest from) the pit in ArcMap 10.4. In each case brighter regions where distortions were expected were avoided when taking these transects. A line was interpolated across the primary pit to these locations and the difference in height between the original bed surface and the depression formed by the CO_2_ ice block was determined. An average and standard deviation of these values were taken in each case and standard deviations fall within the uncertainty margins outlined in *Supplementary Material, Digital Elevation Model Uncertainty Estimates*. Levées were measured in a similar manner by taking average values of the difference between the average flat surface and levée height. Maximum and minimum levée height were recorded in each case.

The area of furrows produced on each bed surface was recorded using polygonal mapping in ArcMap 10.4. The area of the flat pit floor in each case was determined by zooming in to optimal pixel resolution on the orthophoto overlain above the DEM and using the free-hand tool to mark the line where the inner slope of levées ended and the flat pit floor began. The pit floor is defined as the reasonably flat area directly below where the incident block was for which the perimeter is identified as the line between where the inner levée slope ended at 1 pixel resolution. The area of the space between furrow networks was determined by zooming in to optimal pixel resolution and mapping the outer edge of each furrow. This total area was differenced from the total pit floor area to get the area covered by furrow networks. The area covered by furrows was then expressed as a percentage of the total pit floor area. A complete discussion of the DEM uncertainties presented in this paper is available in *Supplementary Material*.

### Data Availability

The datasets generated and analysed during the current study are available from the corresponding author on request.

## Results and Discussion

### Primary Pit and Levée Formation via CO_2_ Ice Block Sublimation

Pits, which are visually comparable to Martian linear gully primary terminal pits, were observed to form via both the (i) stationary and (ii) sliding interaction between sublimating CO_2_ ice and substrate of all grain sizes. The primary terminal pits which punctuate linear gully channels on Mars are characterised by depressions in the substrate encircled by positive relief levées. The pits observed in our laboratory experiments were negative relief features surrounded by positive relief levées which formed primarily by excavation of material via sublimation. Pits were deepest for finer grain sizes (Table 1).

**Figure 3:**
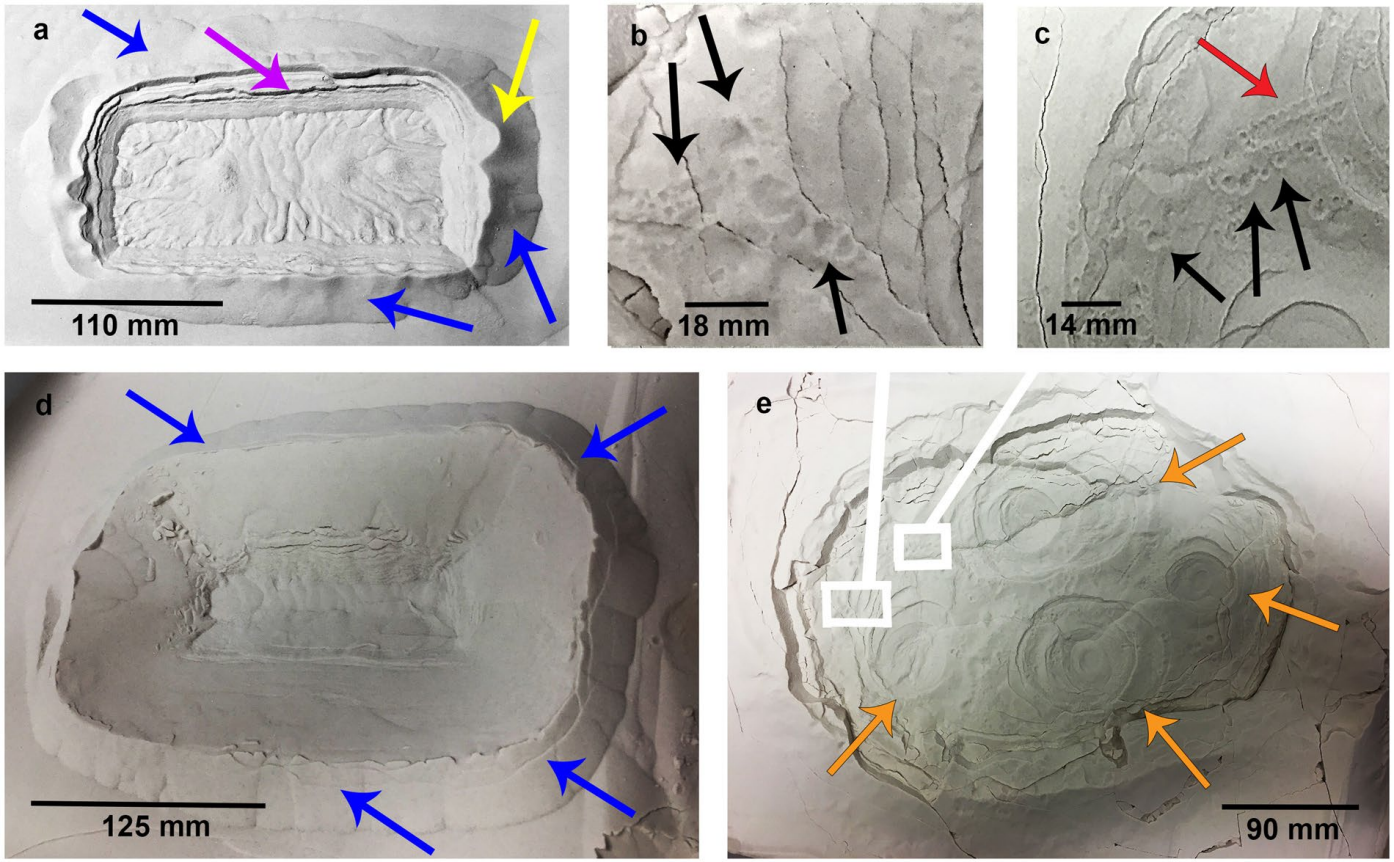
Primary pits and detached impact pits formed during our experiments by the interaction between sublimating CO_2_ ice and granular substrate. **(a)** Primary pit formed by block placement on a bed of 45–90 *μm* grains. Levées are denoted by blue arrows. Yellow arrow points to a preserved vent aperture. Purple arrow indicates a zone of slumping . **(b,c)** Detached impact pits (black arrows) formed by return of granular clusters to a 4–45 *μm* bed surface as a sliding block burrowed beneath the bed and exhibited jet activity from the subsurface. Red arrow indicates a linear string of impact pits. These impact pits were ubiquitous over the primary pit surface (seen in **e**). **(d)** Primary pit formed in another instance of block placement on a bed of 45-90 *µm* grains. Blue arrows point to levées. (**e**) Wider view of the system formed when a block was slid onto a bed of 4–45 *μm* grains. Orange arrows identify collapsed pit morphologies which signify locations where jets emanated from the subsurface. White boxes show locations of **(b)** and **(c)**.

In the cases where material was not transported on top of the block, the resulting pits adopted the shape of the block that formed them (Fig. 3a,d). In our experiments, blocks were rectangular. In other cases, the block burrowed into the bed and superposed material sunk as the block moved downward. In all cases, the primary pit which formed was wider than the block forming it.

Upon initial contact with the substrate which was roughly at room temperature, the block in each case underwent rapid sublimation, thus levitating it and mobilising material within its surroundings. This interaction was interpreted by observation to be brought about by the Leidenfrost Effect, as considered in the CO_2_ block hypothesis. The force of the escaping gas on the grains, transported grains within the sublimating gas from beneath the block to the side, forming levées. During this initial stage of sublimation, vent apertures developed at gaps between the block and the inner slope of levées, from which jets of gas were observed to expel granular material to the main granular sheet (Supplementary movie 1). Some of these vent apertures were preserved after the sublimation process, while others were destroyed by gravitational collapse. After the initial rapid sublimation stage, sediment transport ceased and the block sublimated slowly for ~7–11 hours. It is thought that at this stage, the temperature difference between the block and the granular substrate decreased below the Leidenfrost point, thus ending the levitation and sediment transport phase.

When a block was slid onto the bed surface, a greater range of dynamic responses was observed (Supplementary movie 3). In these cases, particularly in the 4–45 *μm* and 45–90 *μm* grain cases, sublimation dynamics mobilised grains on top of the block. This increased thermal contact between the substrate and the surface area of the block and subjected more grains to the Leidenfrost Effect and the force of rapid sublimation. Pits were generally deeper in these instances, compared to when a block was placed on a bed of the same grain size range (Table 1).

Levées that formed during each experiment increased in maximum height with decreasing grain size (Table 1). This is expected, since decreasing grain size increases grain mobility as the Shields parameter (a non-dimensional number used to calculate the initiation of motion of sediment in a fluid flow^35^), increases. Levée morphologies were comparable to those encircling the terminal pits of linear gullies on Mars. These were raised, positive relief features which surrounded the pit in each case. The relationship between levées and terrestrial debris flows combined with sinuosity has previously been invoked to support a debris flow genesis for gullies (including linear gullies) on Mars^36^. Our experiments highlight the equifinality of the granular response to the movement of dry and wet fluids. Levées formed without the need for downslope block movement and granular material was transported to the side under the influence of pressurised gas alone. This is consistent with the sliding CO_2_ hypothesis which suggests that stationary blocks at linear gully termini are capable of excavating and transporting sediment to form pits and surrounding levées.

### Furrow Formation via CO_2_ Sublimation

**Figure 4:**
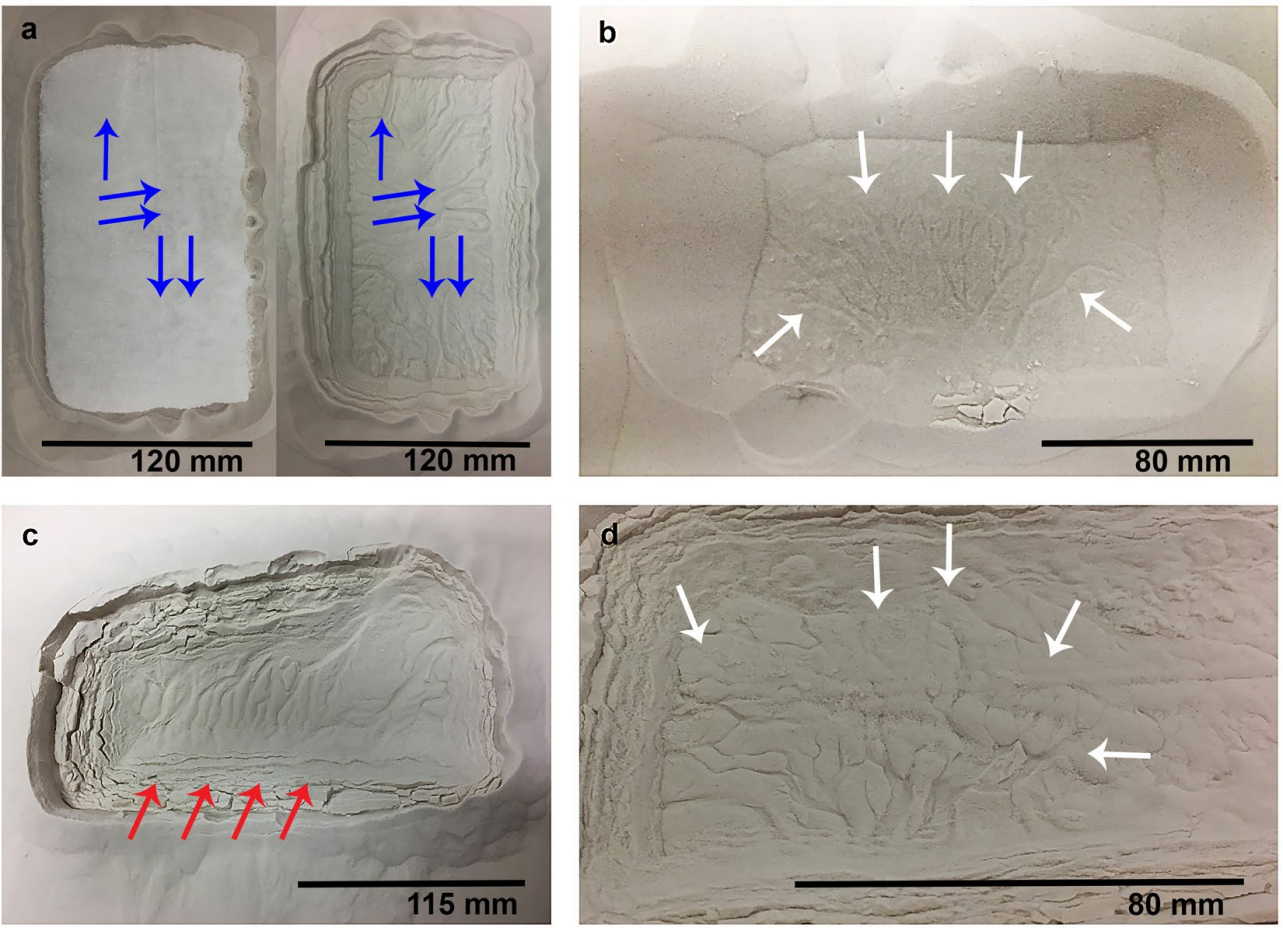
Furrow patterns and networks observed in the laboratory. (**a**) Before (left panel) and after (right panel) sublimation for an experiment on a bed of 45–90 *μm* grains. Left panel: sublimating block. Arrows point to vents where gas escapes from beneath the block. Right panel: primary pit formed on the same bed by the block shown in the left panel, containing linear and sinuous furrows. The direction of gas flow is marked by the convergence of furrows towards vent locations (blue). (**b**) Dendritic network of furrows (white arrows), formed in <1 minute when a block was placed on a bed of 75–150 *μm* grains. This block was lifted and then removed to demonstrate that dendritic patterns can rapidly form. (**c**) Primary pit containing linear and sinuous furrows extending across a pit floor on a bed of 4–45 *μm* grains. Vent apertures were not preserved, however by observation it was noted that vents operated on each side of the block. (**d**) Close-up of dendritic furrow network (white arrows) on a bed of 45–90 *μm* grains.

Our laboratory experiments are the first to show that features similar to the sand furrows on Mars can form by sublimating CO_2_. Furrows formed under the CO_2_ blocks and closely resembled Martian furrows — they were negative relief features similar in pattern and planform though on a smaller scale. The furrows observed displayed a range of patterns (linear, sinuous, dendritic and curvilinear). Martian furrows have been spatially correlated with dark fans, where vents in the ice are posited to form. These fans are thought to be composed of sand transported from beneath the ice by the cryoventing mechanism^12^. Furrow networks observed in the laboratory terminated at vents that developed at the boundary between the ice block and the pit walls (Fig. 4a). When a block was placed on the bed surface and carefully lifted as observed in Supplementary Movie 2, it was discovered that furrow mouths were located at regions where vents formed. Jets at vent locations were observed to transport grains from beneath the block. From these observations, we deduce that it is possible for furrows to be formed by pressurised gas that escapes through vents.

**Figure 5:**
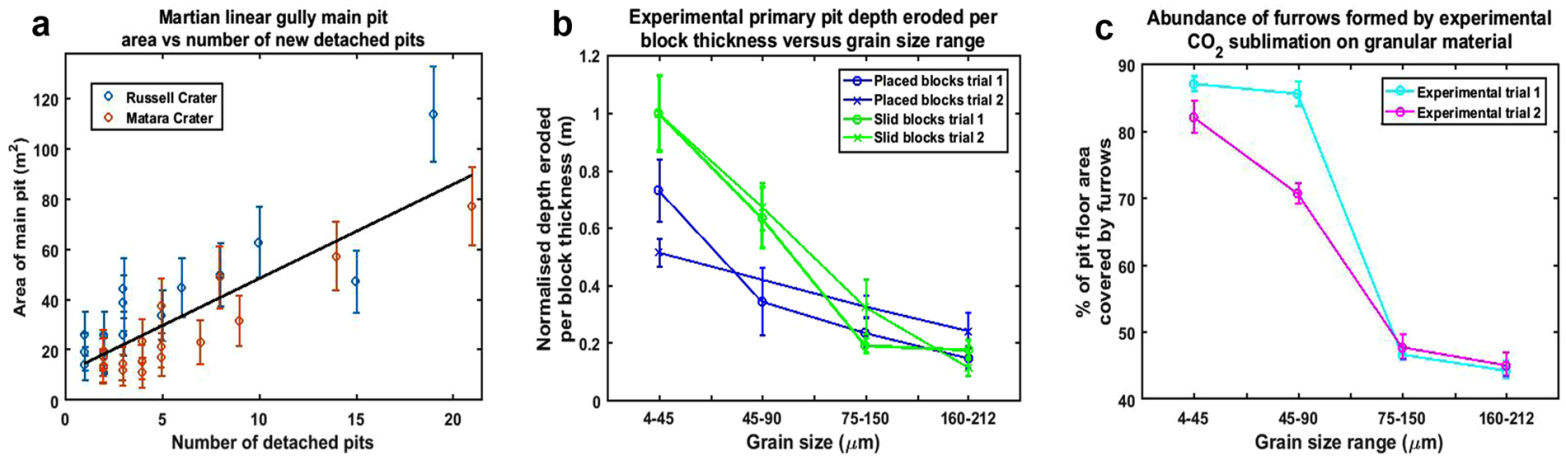
(**a**) Martian Detached pit abundance versus area of terminal pits in Russell and Matara Crater. Plot indicates an increase in the number of new detached pits each spring with increasing linear gully terminal pit area in Russell and Matara Crater. Error bars were calculated by taking 2 × the pixel width for each site and propagating the error on *πr* ^2^ for each main (terminal) pit area). The *R* ^2^ value for the linear fit is 0.72 and the p value is <0.01. (**b**) Laboratory primary pit depth eroded per block thickness. Plot indicating that pits observed in the laboratory were generally deeper on beds of the finest grain sizes. The CO_2_ block placed on a bed of 45-90 grains in experiment 2 partially burrowed and therefore is not included here. Error bars denote vertical error (z error) on each DEM. This was calculated by taking 10 vertical sections of a ruler where available, or known measurement in the scene of the DEM, averaging them and differencing this value from the actual measurement. (**c**) Furrow abundance observed in the laboratory versus grain size plot. Plot shows furrow abundance observed in the laboratory decreased with increasing grain size. Error bars were calculated by defining horizontal uncertainty as better than 1 mm, defining furrows as negative relief erosional lines in the substrate, defining the pit floor as the section at 1 pixel resolution where the inner slope of the surrounding levée ended and relatively flat surface beneath where the block was began, and allowing for error in digitisation decisions when marking the pit floor and furrow outlines in ArcGIS. Considering these factors, uncertainty on measurements was estimated to be 10 orthophoto pixels in each case and their volumes were propagated within the furrow percentage expression to obtain an estimate of uncertainty on furrow abundance.

Furrow abundance increased with decreasing grain size (Fig. 5c). Considering also the general increase in pit depth (Fig. 5b) and maximum levée height with decreasing grain size (Table 1), we report a feedback between grain size and feature formation via CO_2_ sublimation. When grain size distributions on Martian dunes are better constrained, this observation might shed light on why furrows are restricted to certain locations in the southern hemisphere^13^. However, we report that furrow pattern and network type are independent from grain size. Using the furrow pattern and furrow network classification derived from a survey of Martian dunes^12^, we classified the different network types formed by CO_2_ sublimation according to planform. The following classes were delineated: linear (straight negative relief lines in the substrate), curvilinear (curved negative relief lines in the substrate showing one inflection point), sinuous (highly curved negative relief lines in the substrate displaying more than one inflection point) and dendritic (branching negative relief features in the substrate)^12^.

On Mars, the specific location and patterns of furrows on dunes changed between Mars years following emplacement and sublimation of the seasonal CO_2_ ice deposit. We can report a similar outcome for our experiments where furrow location and pattern changes for similar grain sizes between experiments. In some cases, a variety of networks and patterns developed on beds of the same grain size range. We identified dendritic and curvilinear furrow networks on the 4–45 *μm* bed pit floor (Fig. 4d) while only linear and sinuous patterns resulted from a second experiment of block placement on a 4–45 *μm* bed (Fig. 4c).

Dendritic networks were observed in certain experiments across all grain sizes and these developed almost instantaneously as observed in Supplementary Movie 2, rather than via a network growth over time. However, furrows detected in our experiments formed via the contact between a ~−80 °C CO_2_ ice block and substrate which was roughly at room temperature and our incident ice was on a much smaller scale to that which seasonally covers Martian dunes. Our initial conditions were very different to the gradual basal heating of the CO_2_ ice sheet and consequent ice rupture proposed in the cryoventing model. However, we have demonstrated that dendritic patterns can form in a single cryoventing event, as was proposed for Mars^12^.

Dendritic patterns tended to form when there were few vents, and when vents were furthest apart. We note also that when vents were located directly across from one another at each side of the block, linear and sinuous furrows formed instead. This is consistent with the observation that cryoventing efficacy on Mars is constrained by vent spacing^12^. We interpret this laboratory observation as an indication that cryoventing is likely to be limited by a pressure gradient. High pressure gas at the centre of the ice/substrate interface will be attracted towards an area of low pressure — such as that provided by a vent. If there is a roughly equal pressure gradient between the block centre and two lateral vents opposite one another, gas will escape at a similar rate towards either side and this will be reflected in the presence of linear or sinuous patterns that extend and connect across the base of the pit floor. We propose that Martian furrow formation and network type may be influenced by a pressure gradient, supplied either by inhomogeneous ice thickness on dune slopes and/or stress locations such as dune brinks. Further work may clarify the extent to which ice thickness and surface topography influence pressure gradients at furrow and dendritic trough locations on Mars.

### Impact and Collapsed Pit Formation

In addition to primary pit formation, the cases where a block was slid onto grains of 4–45 *μm* and 45–90 *μm* revealed two additional pit types (Fig. 3b,c,e). These instances involved very different fluid dynamics to those observed in all other cases. Grain mobilisation induced by increased thermal contact between ice surface area and the warmer granular material, enabled the block to move downward, and the bed appeared fluidised (Supplementary movie 3). This process lasted ~1 minute in each case before the block was observed to submerge fully within the granular bed. CO_2_ gas jets escaped from the subsurface (Supplementary movie 3), excavating the material in the space surrounding their path and providing room for adjacent material to sink, forming collapsed pits (Fig. 3e). These endogenic pits were characterised by concentric tensional fissures and did not have raised rims.

**Figure 6:**
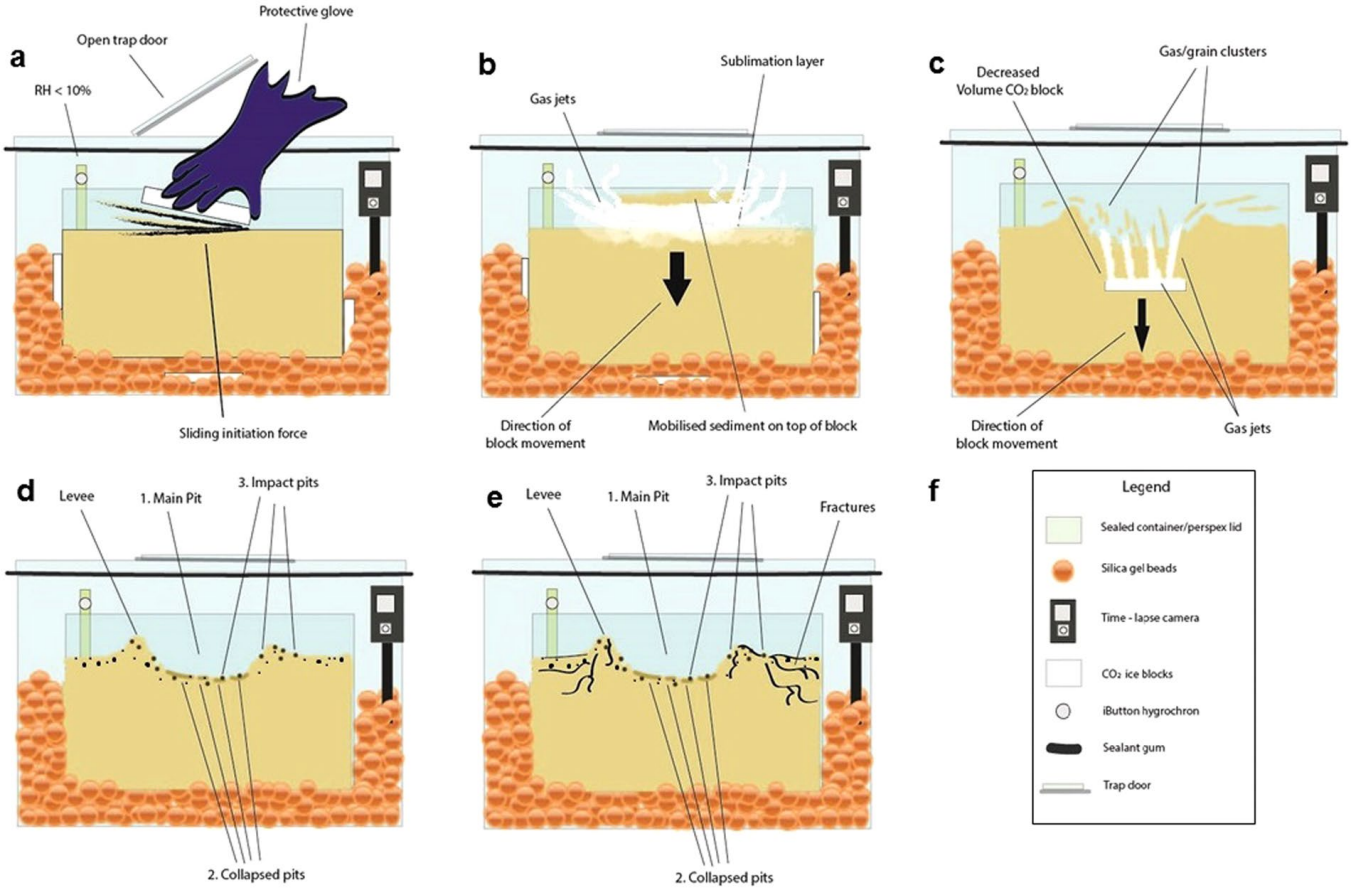
Conceptual model of 3 pit formation modes. (**a**) Block is slid onto grains (beige), increasing thermal contact. (**b**) Grains are mobilised on top of the block via escaping pressurised gas, increasing CO_2_ ice exposure to the Leidenfrost Effect. (**c**) Block submerges; gas jets escape and surface material sinks forming collapsed pits. (**d**) Impact pits form by substrate return via fluid instability such as inelastic collapse of gas jets or granular clustering within jets; returned grains impact the surface forming shallow, rimmed impact pits. (**e**) 3 resultant pit morphologies; primary pit, collapsed pits, impact pits (within main pit and surrounding bed surface) and cracks. (**f**) Legend.

Finally, a third pit type was observed to form via the return of jetted sediment to the steady bed surface. These impact pits (Fig. 3b,c) were smaller in diameter than the primary pit and varied little in size (up to 6 mm in diameter, to below our measurement accuracy). Some occurred in strings and all possessed raised rims, suggesting surface material was ejected in their formation. Following the cessation of sublimation, impact pits were ubiquitous across the primary pit, levées and surrounding bed surface (Fig. 6).

### Pit Measurements in Proctor, Russell and Matara Craters

For morphometric measurements, we surveyed 60 primary terminal pits on Russell Crater megadune and 135 primary terminal pits at 13 sites in Proctor Crater. This preliminary study was carried out purely to demonstrate an initial trend that may help us to assess our laboratory data in the context of Martian dunes and is not intended as an exhaustive survey of Martian linear gully pits.

All terminal pits were characterised by a roughly circular depression surrounded by circular levées, were shallow (<1 m–2.5 m) and varied greatly in diameter, from <1 m to as much as 19 m. Pits were generally wider than their accompanying channels. This is consistent with our laboratory observations that pits were wider in diameter than the sublimating blocks which formed them. We propose that blocks translating down-slope do so quite rapidly and so the width of the channel carved corresponds with block width, while the stationary block may sublimate at the terminus, eroding and transporting material for longer at the site.

In Proctor Crater, the average primary terminal pit width was 3.2 ± 0.5 m and the majority of pits (83.7% of those sampled) were less than 4 m in diameter, with the minimum pit diameter being 1.4 m. The remaining 16.3% of pits were between 4 m and 8 m in diameter (maximum 7.4 m in diameter). Only 16 terminal pits were deeper than the vertical uncertainty of 1.08 m on the Proctor Crater DTM used. The aspect ratios of terminal pits ranged between 0.8 and 1.7.

The Russell Crater terminal pits are larger. Only 11.7% of terminal pits sampled were 4 m or less in diameter, while 46.7% of terminal pits were between 4 and 8 m in diameter and 41.6% were between 8 m and 19 m in diameter. The minimum primary terminal pit width on Russell Crater megadune was 2.7 m and the maximum primary terminal pit width was 18.6 m. Only 3 primary terminal pits were deeper than the upper limit vertical uncertainty of 1.7 m on the Russell Crater DTM, and for these we determined an aspect ratio range between 3.6 and 8.3. Detached pits on Russell Crater megadune of average diameter 2.5 m were found predominantly in the vicinity of the largest primary terminal pits. Other large primary terminal pits were not accompanied by detached pits. However, these particular linear gullies indicated higher probability of block energy loss such as high sinuosity and lateral small channels. These activities may have slowed blocks down or broken them up before reaching the termini.

We observed a trend in Russell Crater and Matara Crater detached pit data (Fig. 5a), which suggests that the abundance of these multiple detached pits at linear gully termini increases with the surface area of the newest/widened terminal pit. Should terminal pits be formed via the sublimation of CO_2_ ice blocks, the data suggest that a greater block size (and hence greater surface area exposed to sublimation) produces more detached pits. This is in agreement with our laboratory observations that “detached” impact pits formed on finer grain sizes when a greater amount of CO_2_ ice surface area was undergoing the initial rapid sublimation dynamics of the Leidenfrost Effect, allowing it to burrow.

We hypothesise that the impact pits observed in the laboratory were formed through a clustering^27^ instability in the granular jets (Fig. 6). This lends credence to our hypothesis that the multiple detached pits surrounding many Martian linear gully terminal pits (Fig. 2a–d) may be formed by a similar mechanism involving granular jets. We reason that at linear gully termini, the sublimating CO_2_ may become unstable as blocks reach a terminal velocity (and greater drag force) at the lower dune slipface, which is reflected in the confinement of these detached pits to the lower ~200 m of the channel vicinity (Fig. 2a–d). Granular clusters may become entrained within sublimating CO_2_ gas jets as the block sublimates. We propose that these are splayed out radially from the terminal pit to form smaller, shallower detached pits upon return impact to the sandy surface, in a similar manner to the mechanism by which impact pits were observed to form in our laboratory experiments (Supplementary movie 4). However, our laboratory experiments required jetting of an endogenic origin which resulted in terraced collapsed pits seen in Fig. 3e. Similar morphologies have yet to be identified on Mars, but it is possible that inset pits seen inside terminal pits may denote locations where blocks have burrowed beneath the surface. Alternatively, inset pits could indicate new, inner paths of blocks within pre-existing channels. However, the ratio of collapsed to impact pits in our laboratory experiments ranged from 5.3–10 and the ratios of inset pits to smallest detached pits in Russell Crater ranged from 4.3–8.1 and so certainly for the smaller detached pits in this setting, our proposed process is plausible. A detailed numerical mode of jetting activity under Martian conditions would allow us to understand the effect of scaling on the proposed process.

In one instance, a high albedo block was measured within a pit on Russell Crater megadune (Fig. 2a) during MY 30, which disappeared the following year and hence was likely to be CO_2_^10^. The pit was observed to widen by 2.1 m in MY 31 during the disappearance of the block. We measured the block in MY 30 to be 2.4 m wide, giving a pit to block widening factor of 0.9. This is consistent with the degree to which we observed pits to erode in the laboratory setting. The pit to block width ratio observed in our laboratory experiments ranged from 1.08 to 1.72. Considering a horizontal uncertainty in this HiRISE image of 0.25 cm/pixel, this observed pit widening by a high albedo block is consistent with a CO_2_ block hypothesis. Considering our scaling arguments, it is likely that the high albedo object was a CO_2_ block which eroded its surroundings to widen a pre-existing pit.

The wide variation in aspect ratio found at both locations is consistent with a dry ice block hypothesis. The blocks that naturally fall from over steepened cornices or steep alcove walls will have a range of sizes and will not be symmetric, thus they will form a wide variety of pit dimensions. Considering the average grain size range reported for the Bagnold dunes on Mars (200–300 *μm*)^32^, we used the values in Table 1 and our calculated scale factor of 0.61 (see Experimental Scaling) to estimate the block sizes needed to form our range of pits. Using our upper grain size ranges of 75–150 *μm* and 160–212 *μm*, we estimate that the block sizes needed to form the pits we have surveyed range from 1.2 m–6.5 m in Proctor Crater and 2.4 m–16.3 m in Russell Crater. High albedo blocks of dimensions between 1.6 and 6 m have been measured in our survey of Russell Crater, however larger blocks have yet to be identified.

The lower range of normalised pit to block width ratios is consistent with dimensions we have observed on Mars using HiRISE images in which high albedo blocks have been identified in linear gully channels in Matara Crater^21^ and in channels and pits in Russell Crater^10^. The ratio of channel width to block width in Matara Crater ranges from 1.9 to 1.1 and in Russell Crater, observed ratios were 1.7 to 2.0. Allowing for measurement uncertainty of one pixel width (0.25 m), these values fall within our estimated range of 1.08–1.72. Although these channels have clearly been eroded prior to the observations of these blocks within them, we can speculate that blocks of similar size to those observed eroded them. Should further data be collected on block sizes at these locations, our laboratory data can be drawn upon to assess whether pit dimensions in the locality are likely to be formed by these CO_2_ ice blocks and perhaps, by how much pit dimensions might grow seasonally.

## Conclusion

Our experiments suggest that furrows and pits can be formed by the sublimation of CO_2_ ice blocks. The differences in temperature, atmospheric density and pressure, and gravity can be accounted for in a scaling analysis by using different sized grains, but the difference in scale cannot easily be dealt with, thus though our evidence is suggestive it is not conclusive. The CO_2_ block hypothesis^21^ is the only current hypothesis that can explain present-day linear gully pit formation. We have shown that stationary sublimating CO_2_ ice blocks in contact with porous, mobile material are capable of transporting sediment to form primary pits and surrounding levées. Our scaling arguments and morphological observations suggest that primary pits may be analogous with Martian primary terminal pits in Russell, Matara and Proctor Craters. In particular, our observation of a high albedo block within a terminal pit and subsequent widening consistent with the ratios predicted from our laboratory data, suggest that linear gully pits may be formed and widened by CO_2_ blocks.

Additionally, we have observed that collapsed pits and detached impact pits; two ancillary pit types, can form by sublimating CO_2_ ice blocks and subsurface jetting. We have presented a new hypothesis for detached pit formation at linear gully termini. This hypothesis is consistent with the observed relationship between terminal pit area and number of associated new detached pits forming seasonally in Russell and Matara Crater and is supported by the laboratory observation that jetting activity can result in impact pits. Further work is required to appropriately address size scaling, and a detailed numerical model may give further insight.

Cryoventing is the only hypothesis that has been offered for sand furrows on Mars, but prior to this study there has been no physical evidence that a pressurised gas layer at a CO_2_ ice and sandy interface can form the complex patterns similar to those observed on Martian dunes. Our results suggest that sublimating CO_2_ generates gas flows sufficiently powerful to mobilise grains on top of the incident ice and add to the granular sheet via venting — a process which is hypothesised to form the dark fans accompanying sand furrows^12^. We have shown for the first time, that a range of furrow morphologies can form by escaping pressurised gas at the interface between a granular surface and a CO_2_ ice overburden, and that furrow pattern type is independent from the grain size distribution (within our grain size range) of the material into which it is eroded. The data suggest that cryoventing efficacy and furrow pattern type are limited by a pressure gradient provided by vent spacing. Additional data is required to assess the role of ice thickness and vent geometry in furrow formation.

Our study has delivered for the first time, physical evidence that (1) linear gully pits and (2) furrows; both active features observed to fade, extend and form in the contemporary Martian climate, may be forming via the action of sublimating CO_2_ ice.

## Electronic supplementary material

Supplementary Material

Supplementary Movie 1

Supplementary Movie 2

Supplementary Movie 3

Supplementary Movie 4
