# Supplementary material for: Experiments On Sublimating Carbon Dioxide Ice And Implications For Contemporary Surface Processes On Mars
Source: Sci Rep. 2017 Oct 27;7:14181. doi: 10.1038/s41598-017-14132-2 (PMC5660181; doi:10.1038/s41598-017-14132-2)
Supplement: Supplementary file 1 — Supplementary Material [file 41598_2017_14132_MOESM1_ESM.pdf]

Supplementary Material: Experiments On Sublimating Carbon Dioxide Ice And Implications For Contemporary Surface Processes On Mars

Mc Keown, L. E.[1,*], Bourke, M. C.[1,2], and McElwaine, J. N.[2,3]

[1]Department of Geography, Trinity College Dublin, Dublin, Ireland
[2]Planetary Science Institute, Tucson, U.S.A.
[3]Department of Earth Sciences, Durham University, Durham, U.K.

ABSTRACT

This document provides supplementary movies, uncertainty analysis, experimental scaling, further calculations, a supplementary figure and tables of parameters referred to in “Experiments On Sublimating Carbon Dioxide Ice And Implications For Contemporary Surface Processes On Mars”.

Supplementary movies

Movie 1 – Vents adding to sheet

Movie highlighting the mechanisms by which gas venting added to the granular sheet and transported sediment on top of a block which was placed on a bed of 4–45 μm grains.

Movie 2 – Furrows forming at vent locations

Movie showing that dendritic furrow network mouths correspond with vent locations. This particular movie shows a block being placed and removed on a bed of 75–150 μm grains.

Movie 3 – Burrowing CO_2 block

Movie showing the submersion of a CO_2 ice block beneath a bed of 4–45 μm glass spheres. Note gas jet activity and consequent grain transport.

Movie 4 – Impact pits forming

Movie showing the formation of detached and linear impact pits by granular clustering within gas jets when a block was slid onto a bed of 4-45 μm grains.

Supplementary Uncertainty Analysis

Digital Elevation Model Uncertainty Estimates

There are several factors which contribute to uncertainties in our DEM measurements. Firstly there are errors due to imprecision in construction and measurement of our scale objects (calibration targets). The construction of the objects was much more accurate than the measurement of their locations which we estimate as better than ± 1 mm. Additionally, changes in relative humidity in the laboratory environment, though small, may have caused the wooden triangle to expand and contract slightly during imaging and external vibrational disturbances cannot be entirely accounted for. We evaluated these errors in the SfM model using a bootstrap approach. We randomly perturbed the measurements of the calibration targets by ± 1 mm and then re-ran the SfM software. As one would expect, if enough images are used and the targets well located, the outputs of the SfM varied by no more than ± 1 mm in each direction.

Although care was taken to sample the flat bed far enough away from the pit site for grain transport to affect the initial surface measurement when measuring pit depths, sediment transport during venting may have introduced a small uncertainty due to potential vertical accretion of $\sim$2 grains in each case where a block was placed onto the surface and in the cases where the block was slid onto the 75–150 μm and 160–212 μm grains. An upper estimate of 10 grains may have accreted onto the flat surface when a block was slid onto 4–45 μm and 45–90 μm granular bed. However, in all cases these fall within the error estimated for our measurements.

Despite efforts to ensure optimum quality when photographing the region of interest, external factors such as the response of the high albedo grains to lighting differences and reflection from the container in some cases, may have led to noise affecting our measurements, particularly in the flat regions used to measure pit depth. We estimated the contribution of noise to our uncertainties in each case by drawing 5 topographic profiles of ∼10 cm very closely together across a flat region of interest. We detrended and averaged this data and estimated noise by calculating the standard deviation in each case. The uncertainty provided by noise is included in Table 1. Additionally, despite zooming to one pixel to take our vertical and horizontal measurements, operator error may have occurred. Having tested our approach by making repeat measurements, we estimate the contribution of this error to be ±1 pixel in each case.

For a pilot run of the first trial of experiments, we included an additional scale which was established by placing a plastic ruler with millimetre graduations on the inner wall of the container which was visible in multiple overlapping images. This scale was established by placing a marker at the lower edge of the marking specifying 0 cm and another at the lower edge of the marking which denoted 30 cm on the ruler and then specifying a scale bar between them of 30 cm. However with this approach, resolution and reprojection error did not differ significantly from models developed having used only the coded markers (Supplementary Material). Additionally, difficulty in placing the points accurately could have introduced auxiliary errors, whereas 12-bit coded markers can be decoded precisely by Agisoft Photoscan. Therefore, we consider automatic coded target detection as more precise than manual marker placement. For the first run of experiments the ruler was reconstructed within the scene of each 3D model but we did not use it for scale bar development. However, the ruler was later used to estimate the vertical uncertainties in the model. For this, a known vertical section of the ruler of 1.3 cm was measured within the model ten times along its length in order to sample any vertical distortions or variations which may have affected our measurements of the nearby pit. For each of these measurements, we zoomed into one pixel and used the Interpolate Line tool in ArcGIS to calculate the vertical distance between the tip of the ruler and a dark strip at its centre which we knew measured 1.3 cm. For the second run of experiments, the ruler was not available within the reconstructed scene. Instead a known distance of 3.3 cm from the top of the container to a horizontal indentation which ran around the inner wall of the container, was measured 10 times along its length within the model in the same manner as for the ruler. The standard deviation of the values from the actual value were the greatest errors involved in our measurements and we have used these as an approximation of z error within our models. These data are included in Table 1.

Supplementary Experimental Scaling Discussion

A complete discussion of the scaling is beyond the scope of this paper and requires a detailed mathematical model. Such an analysis for the levitation of blocks has been presented previously[1]. For the geomorphological effects we build on this work and apply dimensional analysis. The key observation is that behaviour is extremely sensitive to grain size. On Earth and even on Mars, grains with radius $r < 1$ mm are roughly in the Stokes regime and have fall velocity

$$u_f = \frac{2}{9}\frac{\rho_s}{\nu} g r^2, \tag{1}$$

where ρ_s is the density of the particles, ν the dynamic viscosity of the atmosphere and g gravity. ρ_s and ν are roughly the same on Earth and Mars despite the huge difference in atmospheric density. To compensate for the difference in gravity the particles need only be reduced in size by the ratio $\sqrt{g_T/g_M} = \sqrt{9.8/3.7} = 0.61$. The thermal response is given by[1]

$$(T - T_s)I/\sqrt{t}, \tag{2}$$

where T is the initial temperature of the sand, T_s is the sublimation temperature, t is time and I is the thermal inertia. On Mars $I \approx 120\,\mathrm{J\,m^{-2}K^{-1}s^{-1/2}}$ whereas for the glass spheres on Earth $I \approx 420\,\mathrm{J\,m^{-2}K^{-1}s^{-1/2}}$. There is a difference in composition of the granular material used in these experiments and aeolian sand on Mars. Data on chemical composition of grains on Martian sand dunes does not yet appear to be well-constrained. However, preliminary data of grains on the Bagnold dunes[2] reports that grains are mostly dark grey and sometimes show multicoloured patches suggestive of multiple mineralogies. Some are brick-red/brown and may be pieces of local vein fill minerals. Others are green, brown (suggested to be olivine), colourless, and translucent spheres[2]. The grains used in our experiments were soda lime glass beads containing $< 75\%$ SiO_2 and traces of Na_2O, CaO, MgO and free iron. Other environmental differences include Martian atmospheric surface density which is $\sim 0.020\,\mathrm{kg\,m^{-3}}$ whereas Earth's is $1.2\,\mathrm{kg\,m^{-3}}$. Differences in temperatures and atmospheric pressure as well as other differences in sediment properties can be found in (Supplementary table 1). There is also a difference in the sublimation temperatures with $T_s = 147$ K on Mars and 195 K on Earth. Gully activity in the southern hemisphere is thought to have occurred when dune surface temperatures ranged from 190 K to 260 K[3]. The temperature of the granular material used in the experiments was 295 K, but was not precisely controlled and may have been as much as 20 K warmer or colder, ranging from 293 K to 313 K. For our qualitative results the temperature difference was therefore roughly equivalent. The significant difference in thermal inertia

simply means that there will have been a significant heat flux for a $(I_T/I_M)^2 \approx 12$ times longer time. The most significant difference in relation to morphology is the size of the block. On Mars, blocks will be of the order of 1 m whereas blocks used in our experiments were of the order of 0.1 m. A detailed model of grain movement is necessary to account for this, but we have shown that the other differences should not be significant if the grain size is reduced to balance gravity.

Supplementary Calculations

Per communications with S. Sutton in addition to Kirk et al., 2008)[4], we provide calculations of the vertical and horizontal accuracy of the HiRISE DTMs used in our survey of Martian pits.

Horizontal Accuracy

Horizontal accuracy was determined by the post spacing found in the README file of each DTM. For DTEEC_003080_1325_004077 and DTEEC_007018_1255_007229 the horizontal accuracy was 1 m / post.

Vertical Accuracy

Vertical accuracy was determined using the following formula[4]:

$$EP = \frac{\Delta p * IFOV}{parallax/height} \tag{3}$$

Where EP is the expected vertical precision, Δp is the sub - pixel matching quality taken as the total RMS found in the README file of each DTM, IFOV is the Instantaneous Field Of View of the image pixels in meters on the ground. This was determined using the non-map-projected pixel scale of the original data reported on the HiRISE image page under "Original image scale range". The average of the two halves was taken. Parallax/height was taken as the tangent of the convergence angle for each stereo pair (calculated below)

Convergence Angle

The convergence angle is the algebraic sum of the emission angles. If images are taken from opposite sides, emission angles are added. If taken from the same side, these are differenced.

Calculations of Vertical Precision for DTEEC_007018_1255_007229 (Russell Crater)

- Horizontal accuracy: 1 m/pixel
- Δp = 0.372
- PSP_007018_1255 IFOV = 0.254 m
- PSP_007229_1255 IFOV = 0.507 m
- PSP_007018_1255 longitude = 12.9677
- PSP_007018_1255 sub-sc-lon = 13.3769
- PSP_007018_1255 emission angle = 3.42699
- PSP_007229_1255 longitude = 12.9056
- PSP_007229_1255 sub-sc-lon = 12.2307
- PSP_007229_1255 emission angle = 5.73872
- Convergence angle= 3.4+5.7 = 9.1°

$$EP = \frac{(0.372 * 0.507)}{tan(9.1^\circ)} = 1.2m \tag{4}$$

For the calculations of uncertainty on terminal pit area expressed as error bars in Figure 5a, we simply propagated horizontal uncertainty as follows:

$$\sigma A = \sqrt{\Sigma(2\pi r_i)^2 * (\delta_H)^2} \tag{5}$$

where σA is the uncertainty on the area of each pit, r_i is the radius of the primary terminal pit (or additional radii of secondary and tertiary terminal pits) and δH is the horizontal uncertainty on the DTM in each case.

Calculations of Vertical Precision for DTEEC_003800_1325_004077_1325 (Proctor Crater)

- Horizontal accuracy = 1 m/pixel
- Δp = unknown (use 0.2 as an estimate)[4]
- PSP_003800_1325 IFOV = 0.253 m
- PSP_004077_1325 IFOV = 0.282 m
- PSP_003800_1325 longitude = 30.6667
- PSP_003800_1325 sub-sc-lon = 30.1359
- PSP_003800_1325 emission angle = 5.30512
- PSP_004077_1325 longitude = 30.6691
- PSP_004077_1325 sub-sc-lon = 27.664
- PSP_004077_1325 emission angle = 27.7249
- Convergence angle $\sim$ 27.7249-5.30512 = 22.419°

$$EP = \frac{(0.2 * 0.282)}{tan(22.419^\circ)} = 0.15m \tag{6}$$

Since we used an estimate of 0.2 for Δp, the estimate of vertical precision is possibly on the low side. We thus take the estimated vertical precision for this DTM to be better than 50 cm.

References

1. Diniega, S. *et al.* A new dry hypothesis for the formation of Martian linear gullies. *Icarus* **225**, 526–537. DOI: 10.1016/j.icarus.2013.04.006 (2013).

2. Bridges, N. T. *et al.* Investigation of the Bagnold dunes by the Curiosity rover: Overview of initial results from the first study of an active dune field on another planet. In *Lunar and Planetary Science Conference*, vol. 47, 2298 (2016).

3. Raack, J. *et al.* Present-day seasonal gully activity in a south polar pit (Sisyphi Cavi) on Mars. *Icarus* **251**, 226–243. DOI: 10.1016/j.icarus.2014.03.040 (2015).

4. Kirk, R. L. *et al.* Ultrahigh resolution topographic mapping of Mars with MRO HiRISE stereo images: Meter-scale slopes of candidate Phoenix landing sites. *J. Geophys. Res. (Planets)* **113**, E00A24. DOI: 10.1029/2007JE003000 (2008).

Variable	Mars	Earth	Units	Description
		Ambient Conditions		
T_0	260	300	K	Surface temperature
p_0	510	1.01×10^5	Pa	Atmospheric pressure
g	3.7	9.81	$\mathrm{m\,s^{-2}}$	Gravity
		Sand		
κ	0.013	0.26	$\mathrm{W\,m^{-1}\,K^{-1}}$	Thermal conductivity
c	680	830	$\mathrm{J\,kg^{-11}\,K^{-1}}$	Heat capacity
k	10^{-9}	10^{-11}	$\mathrm{m^2}$	Permeability
ρ_s	1 600	1 540	$\mathrm{kg\,m^{-3}}$	Density
$I_s = \sqrt{\kappa c \rho_s}$	119	576	$\mathrm{J\,m^{-2}K^{-1}s^{-1/2}}$	Thermal inertia
		Glass Spheres (0.1 mm)		
κ		0.15	$\mathrm{W\,m^{-1}\,K^{-1}}$	Thermal conductivity
c		800	$\mathrm{J\,kg^{-1}\,K^{-1}}$	Heat capacity
k		3×10^{-11}	$\mathrm{m^2}$	Permeability
ρ_s		1500	$\mathrm{kg\,m^{-3}}$	Density
$I_s = \sqrt{\kappa c \rho_s}$		424	$\mathrm{J\,m^{-2}K^{-1}s^{-1/2}}$	Thermal inertia
		CO_2		
T_s	147	195	K	Ice block temperaturere
e	5.71×10^5	5.71×10^5	$\mathrm{J\,kg^{-1}}$	Enthalpy of sublimation
ν		1.81×10^{-5}	Pa s	Air dynamic viscosity
ν	1.32×10^{-5}	1.37×10^{-5}	Pa s	CO_2 dynamic viscosity
ρ_g	0.010	1.87	$\mathrm{kg\,m^{-3}}$	CO_2 density
ρ_c	1 562	1 562	$\mathrm{kg\,m^{-3}}$	Solid density

Table 1. Relevant physical quantities for Martian Basaltic sand under Martian conditions. (T_0= 260 K and p_0= 510 Pa, dry Navajo Sand under Earth conditions (T_0= 293 K and p_0= 101 kPa) and glass spheres under Earth conditions. Quantities for CO_2 are also specified for Martian and Earth conditions.

**Trial**	**Granular surface temperature (K)**
4–45 μm placed	299
45–90 μm placed	293
75–150 μm placed	301
160–212 μm placed	298
4–45 μm slid	308
45–90 μm slid	298
75–150 μm slid	294
160–212 μm slid	303

Table 2. Granular bed surface temperatures for repeat experiments (trial 2).

Trial	DEM resolution (cm/pix)	Orthophoto resolution (cm/pix)	Target rms reprojection error (pix)	Ruler rms reprojection error (pix)
4–45 μm placed	**0.043**	**0.011**	0.299	0.372
45–90 μm placed	**0.051**	**0.013**	0.230	0.411
75–150 μm placed	**0.051**	**0.011**	0.183	0.264
160–212 μm placed	**0.064**	**0.016**	0.444	0.228
4–45 μm slid	**0.037**	**0.009**	0.253	0.385
45–90 μm slid	**0.039**	**0.010**	1.906	1.352
75–150 μm slid	**0.042**	**0.011**	0.084	0.064
160–212 μm slid	**0.053**	**0.013**	0.236	0.229

Table 3. Model parameters when a ruler was used as an additional reference scale. Because manually placing markers was deemed a less accurate approach, the auxiliary scale was not used for the models reported in this manuscript.

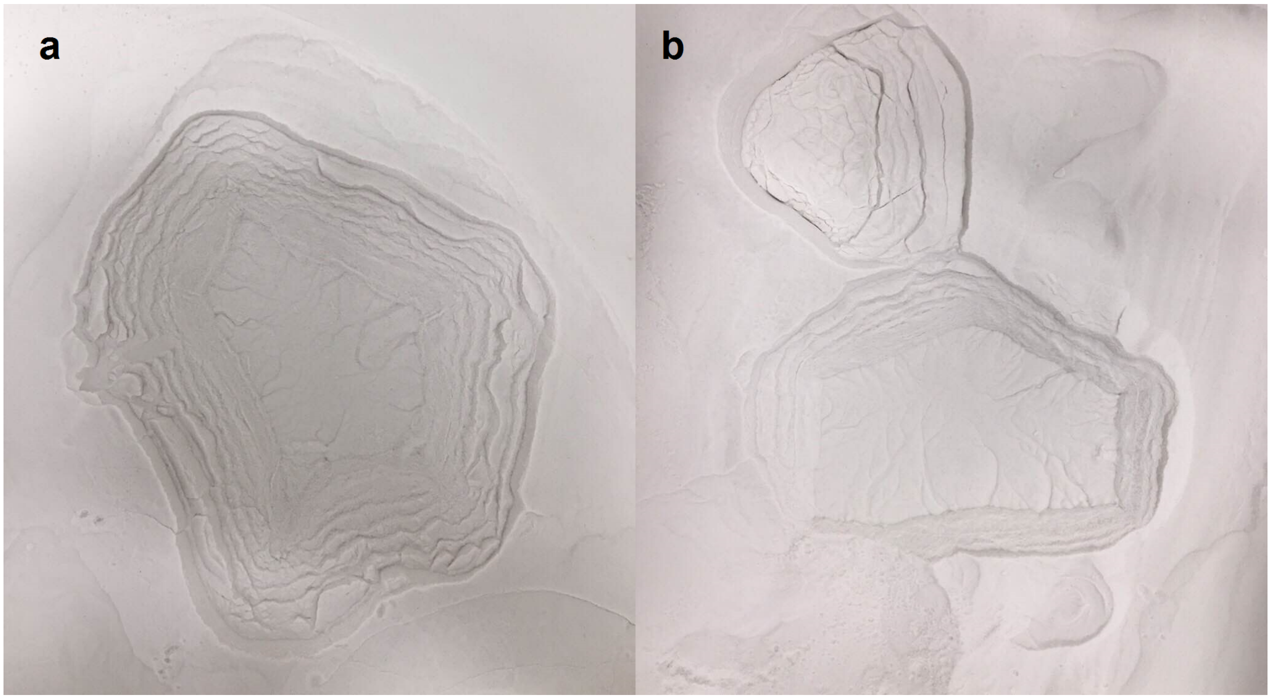

Figure 1. Supplementary Figure: Circular primary pits observed in the laboratory: images showing that circular pits formed when rounded block fragments were placed or slid onto a bed of 4–45 μm grains. (a) shows a pit formed by block fragment placement and (b) shows a pit formed when a slid fragment burrowed (top), juxtaposed with a pit (containing furrows) formed when a block fragment was placed (bottom).
